# Nursing Teachers’ Experiences of Bachelor Thesis Group Supervision: A Qualitative Study

**DOI:** 10.1177/23779608261470906

**Published:** 2026-07-29

**Authors:** Ing-Britt Rydeman, Karin Falk-Brynhildsen, Inger Wätterbjörk, Maria Henricson, Berith Hedberg, Ann-Kristin Isaksson, Birgitta Bisholt

**Affiliations:** 1 Department of Health Care Sciences, 7643 Marie Cederschiöld University, Stockholm, Sweden; 2 Faculty of Medicine and Health, School of Health Sciences, Örebro University, Örebro, Sweden; 3 Faculty of Caring Science, Work Life and Social Welfare, 1802 University of Borås, Borås, Sweden; 4 School of Health and Welfare and The Jönköping Academy for Health and Welfare, 145651 Jönköping University, Jönköping, Sweden; 5 Department of Health Sciences, 7666 Swedish Red Cross University, Huddinge, Sweden

**Keywords:** supervision, nursing students, supervisor, bachelor thesis, experience, qualitative study

## Abstract

**Introduction:**

Supervision of degree theses is a central activity in Higher Education. Writing a Bachelor thesis (BT) is often described as a challenging process for undergraduate nursing students. Previous research on academic supervision has primarily focused on individual aspects at the master’s and doctoral levels rather than nursing teachers’ experiences of BT supervision.

**Objective:**

This study explores nursing teachers’ experiences of Bachelor thesis group supervision in nursing education.

**Methods:**

Semi-structured interviews were conducted with nursing teachers from four universities in the middle of Sweden**.** The data were analysed using reflexive thematic analysis.

**Results:**

The overall theme of the analysis is Different ways of organising and structuring supervision, supported by two subthemes: Finding and developing one’s role as a supervisor and Promoting students’ academic learning. Supervisors shape their roles through guidelines, syllabi, insights from colleagues, co-supervision, and personal experience. Supervisors feel a strong sense of personal responsibility and co-responsibility for their students’ progress. Flexibility is necessary, as not everything can be standardised.

**Conclusion:**

The findings underscore the complexity of the supervisory role and the need for clear organisational frameworks, while flexibility and personal adaptation are key to supporting students effectively. The development of pedagogical supervisory skills is an ongoing process, and organisational support plays a critical role in ensuring supervisors can feel satisfaction and continue to develop in their roles.

## Introduction

Over the last few decades, demands for academic competence in Higher Education have increased, particularly regarding students writing degree projects at various academic levels. The Bologna Process was introduced into Swedish education with a new Higher Education Ordinance in July 2007 (SFS 1993:100) which meant that an independent degree project had to be completed for a bachelor’s degree worth 15 ECTS credits. The scope and goals of this degree project are outlined in the Higher Education Ordinance (SFS 1993:100) and local university policies. The Bachelor thesis (BT) focuses on developing generic research skills and engaging with disciplinary knowledge (Ashwin et al., 2017). Key academic competencies, such as critical thinking, communication, collaboration, and problem-solving are recognised as important learning outcomes of Higher Education (Borglin, 2012). Students are expected to develop essential academic writing and literacy skills, which include approaching topics from a scientific perspective, accessing and interpreting research, evaluating information, and utilising critical thinking (Klarare et al., 2022). These skills should be evident in their degree projects. The degree project is conducted by students under supervision, which plays a vital role in the development of their academic writing and learning. Within nursing education completion of a BT is required. The education leads to both a bachelor’s degree in nursing science and a professional qualification in general nursing. The BT is not compulsory in every faculty or university, but it has many benefits. Students are prepared for their future responsibilities in clinical settings by integrating academic knowledge with practical skills, fostering the foundation for lifelong learning (Millberg-German, 2012). Previous research on academic supervision has often concentrated on master’s (De Kleijn et al., 2012) and doctoral levels (Agricola et al., 2020; Lee, 2008). However, there is limited research focusing on undergraduate supervision, particularly concerning BT (Agricola et al., 2020; Roberts & Seaman, 2018; Todd et al., 2006). Therefore, it is pertinent to investigate how nursing teachers’ experience group supervision regarding nursing students’ BT.

## Review of Literature

Writing a BT is often considered a significant challenge for undergraduate nursing students (Henttonen et al., 2021; Karlsholm et al., 2024; Lundgren & Halvarsson, 2009).The challenges faced by students are partly due to the fact that the content of the BT is not predefined and that they are expected to demonstrate their understanding and knowledge in their chosen BT are (Lundgren & Halvarsson, 2009).

Typically, the BT in Sweden consists of literature reviews based on scientific articles (Johansson & Silén, 2018). This independent degree project occurs in the third and final year of the programme and must meet the criteria for academic writing. Students carry out their degree projects under supervision, where supervisors facilitate academic learning by supporting students to interpret and evaluate information, and to apply in critical thinking (Agricola et al., 2018; Klarare et al., 2022). The best ways to support students in their academic writing during these independent degree projects has been a topic of debate (Jordal et al., 2021). Various supervision models have been proposed to equip students with the necessary skills for their thesis work.

Supervision, whether individual or group-based, is influenced by different pedagogical ideas and traditions at universities. These traditions embody the accumulated experiences of past researchers and educators, along with less reflected but established practices in supervision (Handal & Lauvås, 2005). Traditionally, supervision emphasised the final product—the thesis—within a master-apprentice dynamic focused on developing the research output. In contrast, contemporary focus has shifted toward the learning process. Learning can be understood from various perspectives, including constructivist learning theory, which views knowledge as constructed rather than transferred. This approach emphasises the active role of students as learners, encouraging them to create meaning and build knowledge based on their experiences (Biggs et al., 2011).

Group learning and the concept of peer interaction are not new in Higher Education. Group supervision has also been reported to facilitate collaborative learning, which is recognised as an effective learning method (Cohen, 1994). The interactions among students are crucial, providing opportunities for peer support and peer supervision (Lindgren et al., 2005; Utriainen et al., 2011). Jordal et al. (2021) presents a supervision model designed for two supervisors and group supervision of students working in pairs collaboratively on their degree project. The model follows a step-by-step protocol intended to foster student engagement, responsibility, empowerment and to encourage constructive interaction between students and supervisors throughout the thesis process. An additional model, collective academic supervision (CAS) developed by Nordentoft et al. (2013) for Master´s students, emphasises the relationship between student participation and learning. Furthermore, Agné and Mörkenstam (2018) studied the effect of individual and collective supervision among first-year doctoral students. Their findings indicate that collective supervision increases the likelihood of thesis completion and reduces the time required to complete the thesis. Furthermore, the supervisor’s role within groups is shaped by group dynamics and is characterised by both scientific expertise and significant authority (Holmberg, 2006; Samara, 2006).

BT supervision encompasses both supportive and assessment goals, necessitating guidance while allowing students autonomy and freedom (Strebel et al., 2021). A key component of supervision is the feedback provided by supervisors, which is essential for developing and improving the thesis process. This feedback is closely linked to the relationship between supervisor and student, where various aspects of their interactions and pedagogical approaches are considered (Strebel et al., 2021).

Lundgren and Halvarsson (2009) noted that the supervision process often reflects differing expectations between supervisors and students. Some supervisors find the diversity among students challenging, requiring them to adapt their approaches for each individual (Vehviläinen & Löfström, 2016). Additionally, research indicates that many students in higher education feel unprepared to develop academic texts (Jordal et al., 2021) and struggle with academic writing skills and the academic approach (Borglin, 2012). Nursing students have even expressed concerns about potentially being misled by supervisors as they enter the BT course (Henttonen et al., 2021).

Supervision is characterised as a complex and subtle form of teaching (Agricola et al., 2021). However, most existing studies focus on master’s and doctoral theses, with few examining undergraduate experiences, particularly concerning nursing teachers’ experiences of BT supervision. In the literature search that preceded the study, only a few studies on undergraduate studies and more specifically nursing teachers’ experiences of BT supervision were identified.

## Purpose of the Study

The purpose of the present study was to explore nursing teachers’ experiences of BT group supervision.

## Methods

### Design

The study used a qualitative and exploratory design for gaining a deeper knowledge of nursing teachers’ experiences of nursing student BT group supervision. Semi-structured group interviews were used and analysed using reflexive thematic analysis (Braun & Clarke, 2021).

### Setting

The present study took place at four universities in the middle of Sweden**,** referred to in the results as B (group interviews), C (individual interview), D (group interviews), and K (group interviews). The universities used a group supervision model since 2019 or before to support nursing students writing their BT.

### The Group Supervision Model

The supervision model used at the four universities is organised around themes corresponding to the research process. This group model aims to support students develop a critical and investigative approach, promote active learning, and enhance their ability to work in groups (Perry, 1999). Students typically collaborate with another student to write their BT. The supervision group mainly consists of eight students, organised into four student pairs, along with two supervisors. Each supervisor is assigned to two student pairs, with a specified time allocation of 16 to 20 hours for each BT, in addition to other teaching responsibilities. Supervisors are required to hold at least a master’s degree in nursing.

The meeting schedule includes five sessions at two universities and six sessions at the other two. At two of the universities, each pair has one individual session included. These sessions are mandatory at two universities and take place during the fifth semester (n=3) and the sixth semester (n=1). The course is on a full-time (n=2) or part-time (n=2) basis.

In the very first session, the syllabus, schedule, and supervision model are introduced and discussed, along with the students’ projects. Subsequent sessions consist of two parts: a student-led session followed by a supervisor session. Students are expected to prepare for the student-led session by reviewing drafts from other student pairs and actively discussing and commenting on each other’s texts. This session provides an opportunity to address issues, raise questions, seek resolutions, and set the agenda for the subsequent supervision session. In the upcoming session, students bring up concerns and engage in discussions, while supervisors provide general feedback and outline the focus for the next session, covering topics such as methodology, analysis, and results. Written feedback from the supervisors is also provided to the students.

The final BT is presented at a concluding seminar where students defend their work while a designated student pair reviews and critiques the thesis. The final thesis grade is determined by an examiner (n=3) or a grading committee (n=1) not involved in the supervision process.

### Participants

The participants were recruited through convenience sampling. All teachers supervising BT in the nursing programme at the four universities were eligible to participate (n=73). Written information and an invitation to the study were emailed to all supervisors by a designated contact person at all universities, at one university, the faculty was informed about the study during a workplace meeting. Twenty-one participants took part in the study. The participants had experience supervising nursing students ranging from one semester to ten years. Nine of the participants held master’s degrees, and twelve held doctoral degrees (PhDs). At two of the universities, all participants had master’s degrees.

### Data Collection

The study was conducted during November 2019 to June 2021. Totally seven interviews were conducted (six group and one individual) with 21 supervisors. The number of participants in the group interviews varied between 2-5 participants. The reason there was only one participant in one of the groups was late cancellation. The participant who participated was already booked for the interview. The interviews lasted between 51 to 61 minutes (average 57 min). All were audio recorded and transcribed verbatim by a professional transcription service. Data were processed and stored securely at the servers of one of the included universities, to ensure confidentiality.

Group interviews were chosen to enable supervisors to explore and discuss their views of supervision through group interaction (Kvale, 1996). Two interviews were face-to-face and five took place digitally via Zoom, due to COVID-19 pandemic, by the authors. Interviewers had no connection to the University where the interview was conducted. An observer also participated in the group interviews and took field notes, including key discussion points and noteworthy quotations. The interviews were conducted by all authors and began by giving the same information about the aim of the study to the participants. Semi-structured questions were used, and the questions were as follows: What responsibility do you have as a supervisor in group supervision? Can you describe how you prepare for supervision? What responsibility do you have as a supervisor and the students for their learning? Can you describe how the students learn during the supervision process? What factors contribute to student learning? What is the value for students in writing a BT? What is the value for you in supervising BT? What advantages and challenges do you associate with group supervision? Is there anything else you would like to add that you think is important regarding this topic?

### Data Analysis

**Figure 1.**
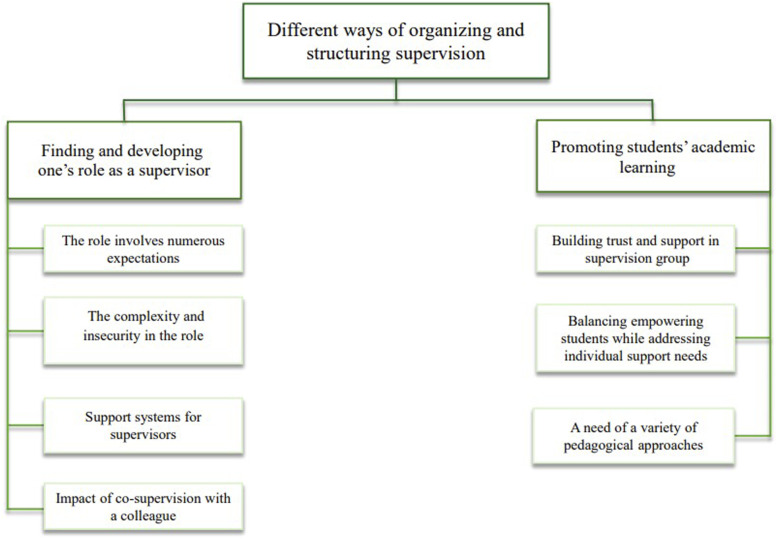
Thematic map presenting the overall theme, sub-themes and codes

The interviews were analysed using reflexive thematic analysis to identify codes, subthemes, and themes (Braun & Clarke, 2021). The analysis process was collaborative, iterative and reflexive to promote a richer and more nuanced understanding of the data. Reflexivity was attained through regular joint discussions by all authors and reflexive questions during the analysis process, the data was iteratively analysed. This meant, we gained insight into our role in interpretation and knowledge production. Reflexivity contributed depth and rigour to the analysis by making our own influences available in data interpretation and theme construction (Braun & Clarke, 2021). The data were manually organised by the first author. The first and last authors read and re-read the transcripts to obtain a deeper understanding of the data and noted ideas and suggestions for the codes. Next, initial codes were generated from the data, and the data relevant to similar codes were collated by the first author. The development of themes was an iterative process, with themes reorganised and refined throughout the ongoing analysis. Themes were constructed by analysing, combining, and comparing how codes related to one another. To gain a deeper understanding of the interconnected nature of the data, a thematic map was created (Figure 1). The thematic map was used to capture the meanings in the codes and facilitated final data reporting. Through the process of creating the thematic map (Figure 1), the complex relationships and interdependencies within the dataset were visually represented.

The steps in the data analysis are presented in Table 1.

**Table 1.** Examples From the Analytical Process

Quote	Code	Sub-theme	Theme
Can I do this? … from a purely rational point of view, there should be no problem. …a great fear of spoiling the students, of missing something important. I know it’s their independent work, so you want them to succeed.	The complexity and insecurity in the role	Finding and developing one´s role as a supervisor	Different ways of organizing and structuring supervision
When you’re a new supervisor, you haven’t had any proper onboarding. You have to jump right in, at least in my case, it took a while before I really understood what I was doing. It’s not always obvious, not even today. It’s quite difficult.	Support systems for supervisors		
As a teacher in a supervision context, I need to work on creating an environment where students dare to ask questions, where everyone has room. I have a responsibility to spread the word, I have a responsibility to give feedback to students that allows them to move forward in their process.	Building trust and support in group supervision	Promoting students’ academic learning	
I supervise in quite different ways. If I have students who are struggling, I’m much more concrete. ‘I think you should do this’ or ‘finish this part.’ I become much more direct and hands-on, whereas for students where things are going smoothly and working really well, I give completely different types of feedback	Balancing empowering students while addressing individual support needs		

### Ethical Considerations

This study was approved by Swedish Ethical Review Authority (Dnr 2019-04774) in Uppsala and follows the Ethical declarations of Helsinki (World Medical Association [WMA], 2024) and the ethical guidelines for research practice (Swedish Research Council, 2024). Institutional permission was obtained from the universities where the interviews were conducted. All participants received written information about the study and were informed that participation was voluntary and that they could withdraw from the study at any time without giving a reason. The participants were also guaranteed confidentiality, and it was explained that their anonymity would be preserved when the findings were presented. Informed consent was obtained orally before the interviews started. The data were stored on the servers of one of the included universities, protected by firewalls, in accordance with the European Union General Data Protection Regulation (GDPR).

## Results

The overall theme of this thematic analysis focuses on Different ways of organising and structuring supervision. This theme is supported by two subthemes: Finding and developing one’s role as a supervisor and Promoting students’ academic learning.

Finding and developing one’s role as a supervisor within the supervisory partner captures the efforts of supervisors to develop their roles through guidelines, syllabi, insights from other colleagues and personal experiences. The dynamic nature of supervisors’ roles also emphasises the critical need for orientation within the supervision context as well as ongoing organisational support, regardless of experience level. It’s crucial for supervisors to understand the guidelines laid out in the syllabus to effectively enforce them. When these elements lack clarity and organisational support is insufficient, insecurity and stress can arise, complicating their role and the supervision process. Supervisors see their role as encompassing various expectations, both in ensuring that students succeed with the BT and in fulfilling their own requirements and responsibilities. Promoting students’ academic learning emphasises the importance of building relationships and creating a supportive and dynamic learning atmosphere in the supervision group. Supervisors emphasise their vital role in establishing security and trust among students to encourage students to express their concerns and seek support when needed. Supervisors emphasise the need to strike a balance between encouraging student independence and academic development while providing essential support for those facing challenges. A dual focus emerges on the importance of guidelines for standardised supervision, while also acknowledging the need for pedagogical flexibility to meet individual student and the group needs, and accommodate diverse educational approaches. When these sub-themes are combined, they highlight the overall theme Different ways of organising and structuring supervision.

### Finding and Developing One’s Role as a Supervisor

The subtheme reflects supervisors’ efforts to explore and develop different educational approaches in supervision. Insights from colleagues, supervisory partners, and their own growing experience are described as crucial for developing appropriate supervisory strategies. Introductory training and ongoing organisational supervision seminars are crucial for developing their role. Supervisors perceive their role as involving multiple expectations, including supporting students in meeting the requirements of academic writing and deepening their understanding of relevant nursing issues, to achieve a passing grade. These expectations are outlined in the syllabus but are also communicated by the supervisors, even though they acknowledge that it is not always possible for all students to meet these expectations.

Last time I had a couple that didn't pass, I've kind of thought about where my responsibility lies, what could I have done differently. I'm new in my role, so yes, I'm learning all the time… I know that it's not me who fails. It's a difficult question, this, about where … where the boundary of responsibility lies, my responsibility and theirs (B4).

In their role as supervisors, they are required to relate to individual students, the group, colleagues, the group supervision model, and the syllabus. The syllabus, grading criteria, and guidelines are described as essential, as they provide a foundational framework and outline the learning outcomes to be achieved. However, when these elements are unclear, they can create confusion in the supervision process.

It's a strange system that just about everyone gets through with a pass on the thesis, very strange. I have supervised some students who I have thought were so bad that I wonder how they can get a pass according to the grading criteria (D4).

The supervisors emphasise the importance of receiving an introduction and support when entering the supervisory role, even for those with a doctoral degree (PhD). As new supervisors, they often feel insecure and express concerns about lacking the competence and knowledge required for the role. This insecurity creates a fear of not being able to adequately support students during supervision. One supervisor noted, “I felt that I lacked knowledge when I started supervising. I was afraid of making mistakes (C1)”.

According to the supervisors, a prerequisite for developing their roles and competencies is the opportunity to discuss supervision with the supervisory partner and at regular organised supervision seminars. Supervision seminars are described as supportive measures that contribute to creating a sense of security in their roles. However, these seminars were not always arranged at their respective universities, and when they were, they often clashed with other scheduled activities due to the supervisors’ commitments to other courses. Additionally, supervisors described challenges in obtaining support from experienced colleagues and in discussing with their supervisory partner, often due to high workload.

The supervisors were organised into pairs, which can influence the supervision process. When two inexperienced supervisors collaborate, it often leads to uncertainty and insecurity, resulting in them spending considerable time preparing and reviewing students’ drafts. Some supervisors benefited from working alongside experienced colleagues, which supported the development of their roles and competencies.

I am new and have supervised together with the course leader this first semester. I perceive her as very structured, and she knows this so well. But now I'm going to change supervision colleague and I'm thinking that I might get a few more suggestions in my toolbox on how to approach a problem. I might learn even more variations from my next colleague (B1).

The supervisors emphasise both advantages and disadvantages to having a fixed supervisory partner versus regularly changing colleagues. Different competencies and experiences within a pair allow them to complement each other. Supervising with the same colleague can provide a sense of security, enhance safety, and promote learning; however, it may also lead to getting stuck in a particular pattern and lacking the necessary pedagogical variety in their approach.

They believe that changing colleagues can facilitate learning and exposure to different supervisory methods, but it can also mean starting anew and experiencing some uncertainty with each transition. Disadvantages mentioned include potential difficulties in collaboration, disagreements on how group supervision should be conducted, or communication breakdowns.

As supervisors gain experience with the group supervision model, their focus shifts from language and details to the overall academic content. One supervisor noted, “I used to be quite focused on the language aspect. Now, with more experience, I pay more attention to the content (B2).”

### Promoting students’ Academic Learning

This subtheme emphasises the importance of establishing relationships with students and creating a supportive atmosphere within the supervision group. The environment is recognised as vital for fostering a sense of security and trust. Group supervision provides opportunities to support students’ learning by addressing different aspects of the research process, fostering constructive group dynamics.

Supervisors highlight from the very beginning that this is an independent endeavour, emphasising that students are responsible for their own learning, writing, and the development of academic reasoning, as well as deepening their knowledge in the field of nursing science. Students’ expectations are identified and discussed, alongside the supervisors’ responsibilities and the course syllabus.

Moreover, supervisors clarify the framework of supervision by emphasising the necessity for students to contribute constructively to the group’s learning and discussions, to make informed choices, and to adhere to text deadlines. They also note that students may encounter difficulties and resistance due to the intensity and demands of the course. “I make students aware that when there is an uphill struggle, difficulties, it is usually part of the process, so that they don't give up when things get difficult (C1).”

The supervisors facilitate group discussions by ensuring that everyone has an opportunity to contribute and to engage in dialogue. Instead of simply answering questions, they encourage students to collaborate and solve problems together. They also address common themes that apply to all student pairs and underline that there is no single correct approach, encouraging students to make justified choices.

When they raise a question, we turn it back to the student group before answering. But how have you thought, how has the reasoning gone? I think this contributes to open discussion and a climate where it is not a question and answer, but a discussion (B4).

As the course progresses, the supervisors’ expectations for student pairs’ independence increase. Supervisors observe that as students systematically work through different parts of their texts, their knowledge and understanding of the academic field improve. Additionally, when students connect the content of their BT with knowledge from previous courses, it contributes to deeper learning, which is often described as a turning point.

It can be challenging when student pairs are not motivated, engaged, or contributing to the development of their thesis. One supervisor stated, “Pedagogically, it’s not possible to teach someone something if they don’t want to (B3).” Nonetheless, the supervisors strive to encourage and motivate students to continue their progress on the BT. They stress the importance of using a variety of pedagogical tools to support student pairs throughout their BT journey, providing examples to ensure that everyone receives the necessary assistance.

The students need different tools to understand. Sometimes it can be good to have extracts from old BT that are good, you can say that this is how you can think and so on. You can assimilate. Others need other types of tools (K3).

When student pairs do not read the comments provided by supervisors or fail to heed feedback, they are encouraged to revisit this feedback. Some student pairs require more support and encouragement; however, supervisors express that they also need to be active participants in the group and take responsibility for their learning. “It’s about adapting the supervision, our responsibility is to support students to achieve their goals. Those who have difficulties I usually give significantly more supervision and individual feedback in the pair (B5).”

Additionally, students who face language difficulties, regardless of their first language, often need significantly more supervision and individual feedback. Supervisors advised these students to make use of the language support services available at the institution, as well as to seek feedback and assistance from peers and friends.

Supervisors frequently find that student pairs resolve their questions during supervision sessions and often ask them to explain their reasoning. Furthermore, they observe that students can identify their own strengths and weaknesses by reviewing and discussing each other's drafts.

Guidelines are considered important for ensuring standardised supervision, while allowing pedagogical freedom to adapt support to each student’s and pair’s unique learning needs and circumstances. The supervisors note that not everything can be standardised, highlighting the need to balance adherence to guidelines with the flexibility to tailor supervision to individual students, each pair and the group.

I do not believe that the group supervision model means I can work freely as a supervisor, we have agreed that it is group supervision, then it applies. But that you can adapt supervision based on the student’s conditions. We have no right as supervisors to say I don’t believe in the model, so I’m running my own race (K2).

## Discussion

The study explored nursing teachers’ experiences of supervision of BT in the final year of nursing education. The supervisors sought to develop their role in promoting students’ academic learning by effectively organising and structuring the group supervision. The findings indicate that expectations of the supervisor role are multifaceted and not entirely clear, posing challenges, particularly for new and inexperienced supervisors. Supervisors faced the dual demands of providing appropriate support to students while ensuring successful BT completion. The need for clear guidelines, a formalised group supervision model and a well-defined syllabus are crucial for supervisors to develop their roles effectively. Organisational factors also play a crucial role in supporting this process.

The responsibility and role of the supervisor are difficult to extract from formal guidelines, as they can vary significantly across different contexts and depend on how supervision is organised. This variation is evident in the present study.

Knowledge and practices related to the supervisory role are not always explicit; much of the role’s content can be implicit and tacit which highlight the complexity of the supervisor’s role. Supervisors reported that they developed their role and strategies through their own experiences and collaboration with their supervisory partner and colleagues. Furthermore, supportive meetings with fellow supervisors were essential for fostering development and collaboration. Despite this, the present study identified a lack of sufficient organisational support and necessary prerequisites for supervisors, particularly for those who are new to the role or co-supervising alongside less experienced colleagues. The complexity of the supervisor’s role is influenced by individual, collegial, and organisational factors, which are crucial for effective academic apprenticeship, as highlighted by Bolinder-Laksov and Scheja (2020).

The findings of the present study, along with previous research, support the notion that the supervisor’s role, even at the undergraduate level, is complex and constitutes a learning process for supervisors themselves. This requires supervisors to be responsive, flexible, and possess strong pedagogical skills. The varied approaches and roles underscore the need for a broad repertoire of skills (Agricola et al., 2021; Utriainen et al., 2011; Ädel et al., 2024).

A study by Ädel et al. (2024) explored the roles of supervisors in BT supervision. A variety of roles were identified, reflecting the different functions within the supervisory process. The coach and counsellor roles emphasise the interactional dimension. The supporting and educator roles serve both institutional and transactional functions. The transactional dimension also includes roles such as subject expert, official, quality controller, professional, and inspector. However, a careful balance in the supervisor’s level of control, and the importance of being perceived as highly supportive should also be taken into consideration (De Kleijn et al., 2012).

Studies report that balancing different roles inherent in supervision presents a challenge for many supervisors (Agricola et al., 2018; Karlsholm et al., 2024; Vehviläinen & Löfström, 2016). Both students and supervisors are used to a one-to-one supervisory relationship and not always prepared for different modes of participation and learning (Nordentoft et al., 2013). Their study highlights the need for enhanced supervisor training in facilitating collective (group) supervision processes.

The results indicated that supervisors felt significant expectations placed upon them, along with a strong personal responsibility for their students’ success. Supervisors are accountable not only to the academic field but also to students, ensuring that their work meets certain quality standards (Olsson & Hallberg, 2018). Supervisors expressed a perceived sense of accountability, often feeling that when a student failed, it reflected a personal failure on their part due to inadequate support. These expectations and feelings of responsibility can be understood through sociological role theory (Biddle, 1986; Merton, 1957), which examines features of social life, characteristic behaviour patterns, and roles. According to this theory, roles encompass sets of expectations, behaviours, and responsibilities associated with a particular position or status within society or an organisation (Biddle, 1986). In this view, the role of the supervisor is perceived as a set of different demands and expectations that must be fulfilled.

The expectations associated with the supervisors’ roles in the present study also encompass not just responsibility but also obligations shaped by organisational factors and the learning environment. Organisational introduction and ongoing support are crucial in preparing supervisors for their roles, particularly when they are new and inexperienced. Supervisors perceived these role expectations as both explicit and implicit, which sometimes led to uncertainty, challenges, and conflicts. They experienced role strain while navigating multiple, often contradictory expectations, resulting in feelings of stress, guilt over perceived inadequacies, and a sense of not performing to the expected standards. Research by Anderson et al. (2006) found that supervisors frequently felt divided in their roles as gatekeepers, tasked with instilling the high academic standards expected of students. Ädel et al. (2024) also highlighted the need for a careful balance in the supervisor’s control, and the importance of being perceived as highly supportive. For those new to the supervisory role, adapting to prevailing expectations and norms proved challenging, particularly in the absence of organisational support. The findings also underscore the importance of establishing a strong supervisory relationship to promote student learning. This involves creating a supportive learning environment where students feel included and safe, while also clarifying the framework for the course and supervision. Ädel et al. (2024) and Roberts and Seaman (2018) underscore the importance of discussing expectations and roles early in the supervisory process to foster awareness and align attitudes regarding supervision. Clarifying expectations is key to building a successful supervisory relationship and can help mitigate unrealistic expectations from both supervisors and students. Differing expectations can disrupt the supervisory process and significantly detract from the overall experience for both parties (Roberts & Seaman, 2018).

As the BT progresses, supervisors gradually increase their expectations for student independence, adapting their support and approach throughout the BT process. When students share diverse perspectives, it enhances various approaches to problem-solving. Other studies confirm that adapting supervisory strategies to meet students’ needs and encouraging them to think independently, positively impacts their learning and the progress of their BT (Agricola et al., 2021). Promoting active and constructive learning, self-directed learning, and collaborative engagement by supervisors contributes to enhanced problem-solving quality and overall group functioning (Dysthe et al., 2006).

The findings indicate that relying on a single role or approach in supervision is insufficient. With growing experience, supervisors refined their supervisory practices. Standardised models can provide a framework, but supervisors also need pedagogical freedom to adapt to the varying learning needs of students. The importance of having the flexibility to move from standardised strategies to more tailored pedagogical approaches, especially when students require additional support, is emphasised by Ädel et al. (2024).

The findings highlight differences in students’ levels of knowledge, personal abilities, and motivation, which can present challenges for supervisors. There is often uncertainty about how to effectively meet the diverse needs of students. Since most undergraduate students lack research experience, it is essential that these skills are developed throughout their education and work on the BT (Agricola et al., 2018). The nursing program BT is a critical academic requirement, representing students’ first major final work on an academic education and contributing to both their academic degree and professional qualification. Supervisors in this study described the course as demanding, noting that students often encounter difficulties. Other research indicates that students in Higher Education are not always adequately prepared to develop and write academic texts (Jordal et al., 2021; Lundgren & Halvarsson, 2009). We therefore argue that students should be provided with early and continuous opportunities throughout their education to develop essential academic writing, information evaluation, and critical thinking skills. Undergraduate thesis in nursing education are valuable for students’ personal and professional development. Nursing education is expected to prepare students to become consumers of research, an essential skill for ensuring safe patient care (Aiken et al., 2014), while also fostering their potential to develop as researchers themselves.

## Strengths and Limitations

The participants included in the study are from the nursing education programmes of four universities. These programmes include a BT of 15 European Higher Education Credits and group supervision. It can be a limitation that the length of experience varies among the participants, and the majority have a PhD degree. However, it does not appear in the interview data that the experience of the role differs.

The plan was to conduct focus group interviews with at least five participants at each university, but this had to be changed as fewer participants indicated their interest in participating. This affected group size, and one group consisted merely of an individual interview. The number of participants in the groups can be seen as a limitation, but saturation was discussed during the analysis process and was assessed to be reached after analysis of all interviews. In a focus group interview, some participants may dominate the conversation, while others may feel uncomfortable expressing their opinions (Freeman, 2006). The observer’s participation in this study was important to ensure that all participants spoke. The limitation was that this individual interview did not provide opportunities to share experiences with others. The interviews were conducted digitally via Zoom due to the ongoing COVID-19 pandemic. It may have affected the ability to share experiences and capture nuances that may have been lost. We have found that data from group interviews is a useful method for collecting data that captures how supervisors experience their role in the context in which they are involved.

The reflexivity contributed depth and rigour to the analysis and theme construction. The combination of a semantic and critical approach in the analysis was significant to facilitate a reflexive analysis. It has been important to take a critical approach and to carefully and reflexivity consider the interaction between researchers, the data and the social context being studied (Braun & Clarke, 2021). However, the data provide an understanding of nurse educators’ experiences of group supervision of students’ BT in their final year of nursing education.

## Implications for Practice

This study indicates that the supervisor’s role in the context of BT supervision is a complex and dynamic process that involves the organisation and structuring of the supervision process. Higher Education must support and provide teachers with the necessary conditions in the development of the supervisor role so that they can meet the needs of each student in their academic learning and development. An important contribution based on the results is to offer regular and continuous support in Higher Education, opportunities for professional development and access to clear guidelines and syllabi. Based on the results, group supervision is a suitable method for promoting academic learning and supervising BT in Higher Education.

## Conclusions

The findings highlight the complexity of the supervisor’s role in supervision, which is shaped by a mix of individual, collegial, and organisational factors. Supervision at the undergraduate level is described as a dynamic, evolving learning process for supervisors, where they improve with experience. However, this development can be significantly hindered without adequate organisational support. The study emphasises the importance of the reciprocal relationship between supervisors’ development and organisational support structures. Despite these challenges, supervisors feel a strong personal responsibility for their students’ progress and sense of co-responsibility for their success. Establishing relationships, creating a supportive and inclusive learning environment, and clarifying responsibilities are vital for students’ success. Supervisors must continuously adapt their approach and develop a broad range of pedagogical skills to meet the diverse needs of their students. A flexible approach is necessary, as not everything can be standardised. This balance between standardisation and flexibility is crucial for addressing both the needs of supervision groups and those of individual students in the learning process.

## Data Availability

The interviews and the analysis are available in Swedish language. Examples of the analytical process are shown in Table 1.*
